# Combined double tarsal wedge osteotomy and transcuneiform osteotomy for correction of resistant club foot deformity (the bean-shaped foot)

**DOI:** 10.1007/s11832-015-0650-3

**Published:** 2015-03-20

**Authors:** Roger Jawish

**Affiliations:** Department of Orthopaedic Surgery, Sacré Coeur Hospital, P. O. Box 116, Hazmieh, Lebanon

I read with interest the article “Combined double tarsal wedge osteotomy and transcuneiform osteotomy for correction of resistant club foot deformity (the bean-shaped foot) by Elgeidi and Abulsaad [1]. I would, however, like to draw attention to some errors in their description of the double osteotomy technique.

In their article the authors mention that the procedure described by Jawish [2] in his 1994 article was the sole opening wedge osteotomy of the first cuneiform technique. This is not correct, and in fact in this article the author describes a single medial osteotomy for the foot with primus varus, as well as a double osteotomy for feet with resistant metatarsus adductus observed in the clubfoot and Z-shaped-foot. To quote part of the abstract of Jawish’s [2] article: “in resistant metatarsus adductus, closed wedge osteotomy of the cuboid has been added to correct the varus deformity of the fore foot, it allows lateral swing of the fore foot: the bone excised from the cuboid is use to stabilize medial osteotomy”.

**Fig. 1:**
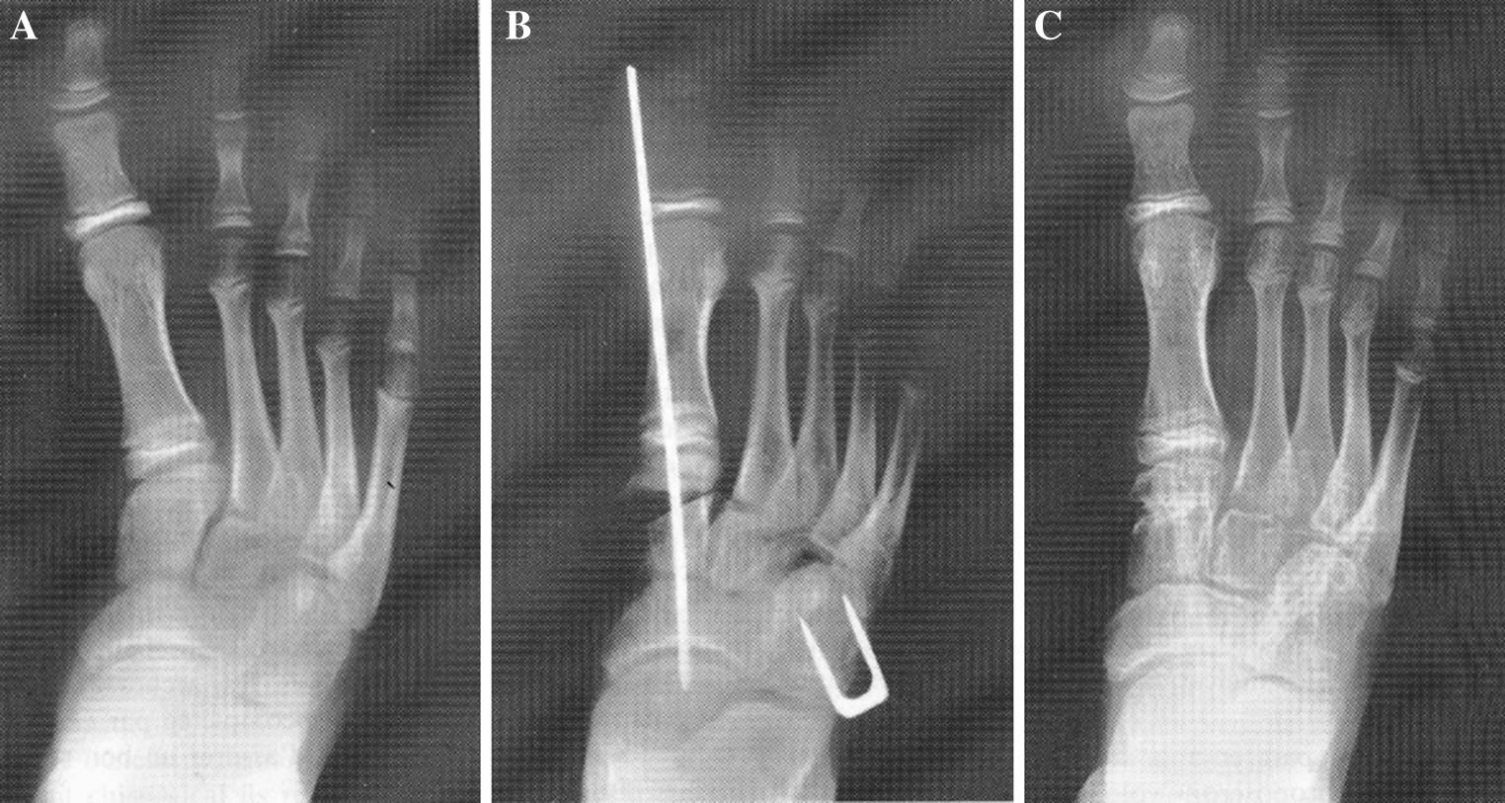
(Boy DEL...Stéphane) **a** At 12 years old, the patient had a Z-shaped foot grade 4 with no initial treatment. He has metatarsus adductus and lateral deviation of the anterior tarse, with deformities of the first cuneiform and the cuboid. The talo-calcaneous angle is normal. **b** After mobilisation of the Lisfranc, we performed a closing wedge osteotomy of the cuboid and opening wedge osteotomy of the first cuneiform allowing good alignment of the first ray. The pins are removed after 2 months, the cast after 3 months (**c**). After one-year follow-up, the clinical correction and radiological aspect remained excellent. This procedure is recommended for the treatment of the Z-shaped foot after the age of 4–6 years (This illustration is from Jawish et al. [4])

In the same paragraph of the article of Elgeidi and Abulsaad [1], the authors report that McHale and Lenhart [3] were the first to describe, in 1991, the double osteotomy (opening of the cuneiform and closing of the cuboid). Again, this is incorrect: the double osteotomy technique was first performed by Jawish and colleagues in Paris (Hôpital Enfants-Malades), and their description of the technique was published in* Chirurgie Orthopédique* (a French-language journal) in 1990 [4]. The double osteotomy technique described by Jawish and colleagues in 1990 was performed to correct the resistant adduction of the forefoot in the Z-shaped foot and was performed exactly as cited in 1991 by McHale and Lenhart for forefoot adduction in club foot. The technique is mentioned in the abstract of the 1990 article [4], and illustrations of the double osteotomy are provided in the article itself, with follow-up (Fig. 1).

To summarize, the double osteotomy of the cuneiform and the cuboid is a technique reported in 1990 by Jawish et al. [4], developed by these authors in the Department of Pediatric Orthopaedics of Hôpital Enfants-Malades (Paris) to treat resistant adduction of forefoot. The article published in 1994 by the first author reported the application of the same technique in a series of children with multiple causes of forefoot deformities.
